# A case of neonatal lupus erythematosus presenting with extensive erosions at birth, healing with extensive scarring

**DOI:** 10.1016/j.jdcr.2023.09.017

**Published:** 2023-10-01

**Authors:** Sheau Yun Kan, Sridhar Arunachalam, Selina Ho, Chong Chia Yin, Thaschawee Arkachaisri, Mark Jean Aan Koh

**Affiliations:** a Department of Dermatology, KK Women’s & Children’s Hospital, Singapore; b Department of Neonatal & Developmental Medicine, Singapore General Hospital, Singapore; c Infectious Disease service, KK Women’s & Children’s Hospital, Singapore; d Rheumatology service, KK Women’s and Children’s Hospital, Singapore

**Keywords:** congenital lupus erythematosus, herpes simplex, neonatal lupus erythematosus

## Introduction

Neonatal lupus erythematosus (NLE) is a rare disorder affecting newborns due to maternal transplacental transfer of autoantibodies. Congenital lupus is even rarer, and this term has been used to describe a subset of neonatal lupus that presents at birth. We report an unusual case of NLE presenting at birth with extensive erosions at pressure-dependent areas.

## Case report

**Fig 1:**
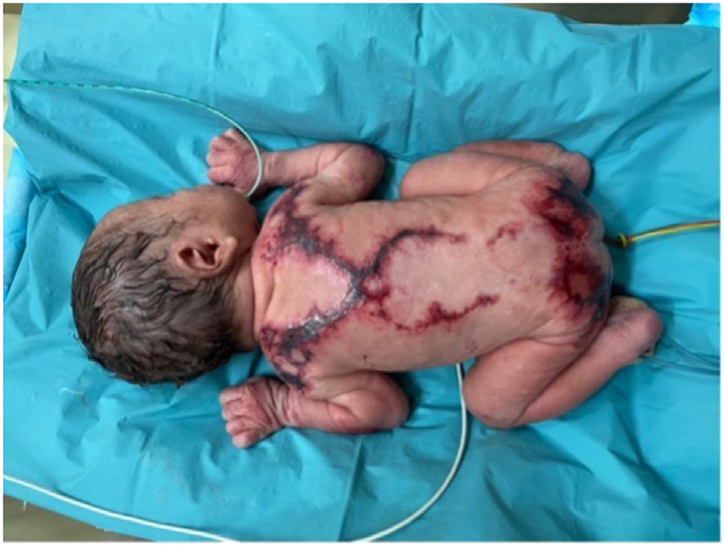
Extensive areas of purpuric erosions over the back, buttocks, anterior aspect of the chest, arms, neck, and scalp were noted at birth.

The patient is a Chinese boy born at 36 + 6 weeks gestation via elective lower segment cesarean section. Upon delivery, he was noted to have extensive areas of purpuric erosions over the back, buttocks, anterior aspect of the chest, arms, neck, and scalp. Interestingly, the erosions on his back formed a characteristic “X”-shaped configuration. His oral mucosa, hair, and nails were normal (Fig 1). He was born flat and dusky, required positive pressure ventilation for 3 minutes, and remained hemodynamically stable after this.

He is the first living child of nonconsanguineous parents. His mother is a 33-year-old female with systemic lupus erythematosus diagnosed at 17 years of age. Her disease control had been relatively stable on oral prednisolone 5 mg daily, hydroxychloroquine 300 mg daily, and aspirin 100 mg daily throughout the pregnancy. There was 1 previous miscarriage, and the current pregnancy was conceived via *in vitro* fertilization. Antenatally, fetal growth, amniotic fluid, and fetal abnormalities scans were unremarkable.

Polymerase chain reaction (PCR) for herpes simplex virus (HSV) type 1 returned positive from surface swabs of the skin lesions on his back and oral cavity, and the HSV total antibody was 1:128 on day 2 of life. Cerebrospinal fluid, blood, and conjunctiva for HSV PCR and serum HSV Immunoglobulin M serology (done on day 2 and day 11 of life) were negative. Ophthalmological examination was normal. Mother did not have a history of oral or genital ulcers, but her HSV Immunoglobulin G serology was 1:128. The baby’s skin lesions were negative for cytomegalovirus PCR, varicella-zoster PCR and *Toxoplasma gondii* PCR. He was started on parenteral acyclovir for 2 weeks and was converted to oral acyclovir thereafter.

Serological evaluation for congenital lupus done on day 1 of life showed antinuclear antibody titer at 1:320 in homogeneous pattern, and anti–double-stranded DNA antibody at 219.35 IU (<25 IU negative). Antiextractable nuclear antigen profile was negative. Electrocardiogram and 2D echocardiogram were normal. Blood count and liver function test were normal. Further investigations were performed to exclude antiphospholipid syndrome, including anticardiolipin antibodies, lupus anticoagulant, anti-β2 glycoprotein 1 antibodies, ultrasound Doppler of kidneys and renal vessels, and cranial ultrasound, which all returned unremarkable.

**Fig 2:**
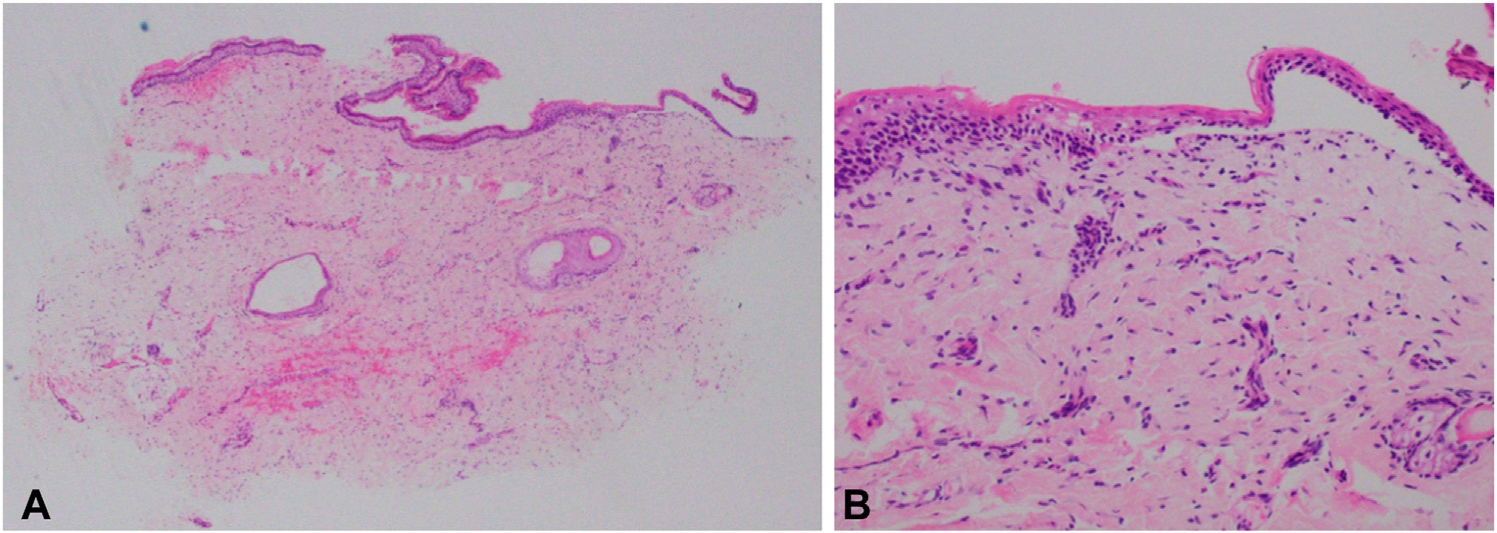
Skin biopsy on a purpuric lesion from the back **(A)** hematoxylin-eosin stain; original magnification: ×40, showing atrophic epidermis lined by mildly compact orthokeratin, with red cell extravasation in the superficial and mid dermis, and **(B)** hematoxylin-eosin stain; original magnification: ×200, showing subepidermal blister with mild basal vacuolar degeneration in the adjacent epidermis.

Skin biopsy (Fig 2) on a purpuric lesion from the back showed a subepidermal blister with basal vacuolar degeneration and some extravasated red blood cells. No viral cytopathic changes were noted. Both HSV1 and HSV2 immunohistochemical stains were negative. Direct immunofluorescence was negative for Immunoglobulin G, Immunoglobulin A, Immunoglobulin M, C3, and fibrin.

**Fig 3:**
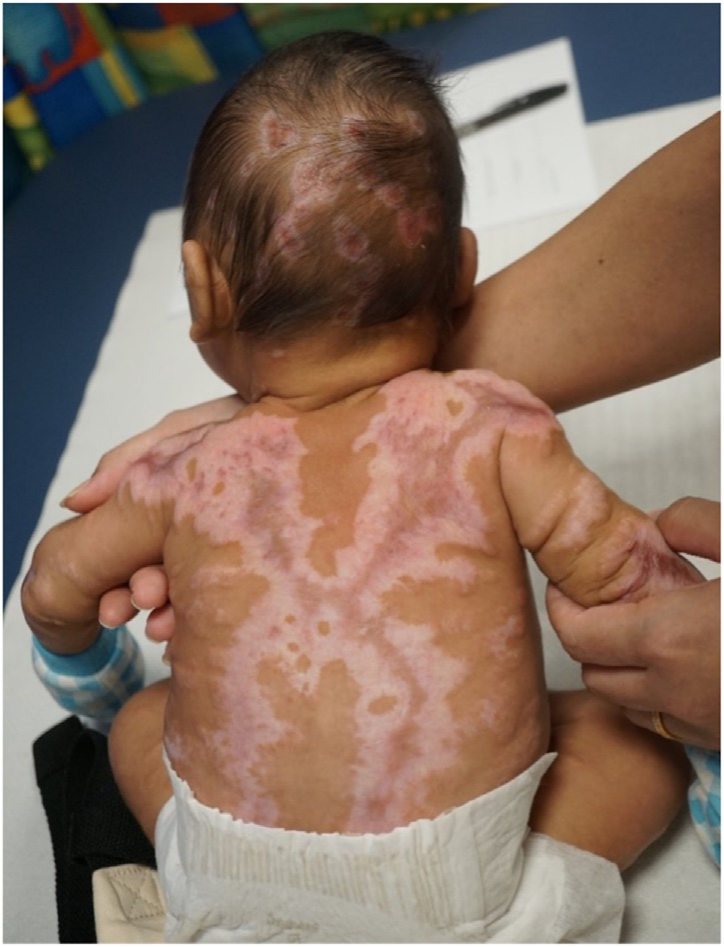
At 3 months old, previous skin lesions healed with atrophic and hypertrophic scars.

The skin lesions healed with atrophic and hypertrophic scars (Fig 3) over the next 3 months with no new vesicular or erosive lesion observed. Alopecia was seen over previously involved scalp areas. In view of decreasing HSV antibody titers, (1:128 on day 2 of life, 1:64 at 2 months old, and 1:32 at 4 months old), oral acyclovir was stopped after 2 months, as he was deemed not to have congenital HSV infection. At the 15-month review, his growth and development were normal. A repeat skin biopsy performed on an atrophic hypopigmented area at 4 months of age showed mild spongiosis in the epidermis, a thinned dermis, and fragmentation of dermal elastic tissue on the Elastic Verhoeff Van Gieson stain, consistent with scarring.

In view of the significant maternal history of systemic lupus erythematosus, with no other possible etiologies found after extensive investigations, and the progression of the erosive lesions to scars, we concluded that the skin lesions at birth were likely secondary to NLE.

## Discussion

NLE is due to passage of maternal antibodies through the placenta into the fetal circulation. Cutaneous manifestations are the commonest presentation of NLE. Other features include heart block, hepatic, hematologic and neurologic manifestations.

Our patient presents several atypical features of NLE. Presentation of NLE is uncommon at birth, as patients usually present after a few weeks of life, following light or sun exposure. The most common presentation is erythematous scaly annular plaques over the face. Our patient presented with extensive purpuric erosions in highly unusual conformations, healing with atrophic, hypopigmented scars. Scarring has been reported to be more common in NLE with cutaneous lesions at birth compared with classical cutaneous lesions of NLE—which are typically nonscarring and resolves by 3 to 6 months.^1^^,^^2^

The distribution of the necrotic lesions at birth appear to correspond to pressure sites of a fetus *in utero* (back, buttock, chin, scalp, sparing anterior aspect of the chest, and face). We postulate the possibility of intrauterine vascular compromise from autoantibodies as an antecedent event, leading to reduced perfusion to these areas of skin, eventually giving rise to erosions secondary to the pressure caused by the uterine wall on the skin.^3^

In addition, only double-stranded DNA was positive in our patient, with negative anti-Ro/SS-A and anti-La/SS-B antibodies. Both anti-Ro/SS-A and anti-La/SS-B antibodies are the most common autoantibodies found in infants with NLE, giving rise to the classical annular rash and associated with congenital heart block, the most severe complication of NLE.^1^

Differential diagnosis of widespread erosive lesions at birth includes infections, eg, congenital HSV infection, varicella-zoster infection, staphylococcal-scalded skin syndrome, neonatal candidiasis; genodermatoses, eg, epidermolysis bullosa, incontinentia pigmenti, and others, eg, focal dermal hypoplasia, aplasia cutis congenita, and autoimmune bullous diseases. Detailed maternal and family history with thorough examination of the skin, mucosal surfaces, hair, and nails should be performed. Skin biopsies may be considered to provide additional information with regards to an underlying etiology.

The prognosis is generally good for babies with NLE, especially the cutaneous manifestations. In classical NLE, lesions typically heal without scarring. However, sequelae such as scarring, dyspigmentation, and telangiectasias can occur, especially in congenital lesions and with extensive involvement. Treatment is usually not required, though topical corticosteroids may hasten the resolution of lesions.^4^ Sun protection is advised. Extensive scarring may result in psychological impairment to patient and parents, and this should be monitored for, and support provided accordingly.

## Conflicts of interest

None disclosed.
